# Diarylpentanoid (1,5-bis(4-hydroxy-3-methoxyphenyl)-1,4-pentadiene-3-one) (MS13) Exhibits Anti-proliferative, Apoptosis Induction and Anti-migration Properties on Androgen-independent Human Prostate Cancer by Targeting Cell Cycle–Apoptosis and PI3K Signalling Pathways

**DOI:** 10.3389/fphar.2021.707335

**Published:** 2021-07-20

**Authors:** Nurul Azwa Abd. Wahab, Faridah Abas, Iekhsan Othman, Rakesh Naidu

**Affiliations:** ^1^ Jeffrey Cheah School of Medicine and Health Science, Monash University Malaysia, Bandar Sunway, Malaysia; ^2^ Laboratory of Natural Products, Faculty of Science, Universiti Putra Malaysia, Serdang, Malaysia; ^3^ Department of Food Science, Faculty of Food Science and Technology, Universiti Putra Malaysia, Serdang, Malaysia

**Keywords:** diarylpentanoid, androgen-independent prostate cancer, anti-cancer, cell cycle, apoptosis, anti-migration, PI3K pathway, gene expression

## Abstract

Diarylpentanoids exhibit a high degree of anti-cancer activity and stability *in vitro* over curcumin in prostate cancer cells. Hence, this study aims to investigate the effects of a diarylpentanoid, 1,5-bis(4-hydroxy-3-methoxyphenyl)-1,4-pentadiene-3-one (MS13) on cytotoxicity, anti-proliferative, apoptosis-inducing, anti-migration properties, and the underlying molecular mechanisms on treated androgen-independent prostate cancer cells, DU 145 and PC-3. A cell viability assay has shown greater cytotoxicity effects of MS13-treated DU 145 cells (EC_50_ 7.57 ± 0.2 µM) and PC-3 cells (EC_50_ 7.80 ± 0.7 µM) compared to curcumin (EC_50_: DU 145; 34.25 ± 2.7 µM and PC-3; 27.77 ± 6.4 µM). In addition, MS13 exhibited significant anti-proliferative activity against AIPC cells compared to curcumin in a dose- and time-dependent manner. Morphological observation, increased caspase-3 activity, and reduced Bcl-2 protein levels in these cells indicated that MS13 induces apoptosis in a time- and dose-dependent. Moreover, MS13 effectively inhibited the migration of DU 145 and PC-3 cells. Our results suggest that cell cycle-apoptosis and PI3K pathways were the topmost significant pathways impacted by MS13 activity. Our findings suggest that MS13 may demonstrate the anti-cancer activity by modulating DEGs associated with the cell cycle-apoptosis and PI3K pathways, thus inhibiting cell proliferation and cell migration as well as inducing apoptosis in AIPC cells.

## Introduction

Prostate cancer (PCa) is the most common malignancy and the second-leading cause of cancer-related death among men worldwide (Crawford, 2003). Androgens and its receptor are the dominant modulators of normal prostate growth and PCa tumorigenesis (Banerjee et al., 2018). Androgen depletion therapy (ADT) is extensively used to treat PCa and effective in controlling hormone-naïve PCa (Huggins and Hodges, 2002). However, over time, these patients eventually progress to a more aggressive and lethal form of PCa, known as androgen-independent PCa (AIPC) (Aragon-Ching and Dahut, 2007). Despite the advancement of PCa therapy, the mortality rate remains high, mainly among AIPC patients (Isaacs, 1994; Fiñones et al., 2013). Furthermore, the effective treatment options for this disease are still lacking, and the drugs available are mostly palliative (Chang, 2007). Therefore, there is an urgent need to identify novel and potent anti-cancer compound to treat AIPC.

**FIGURE 1:**
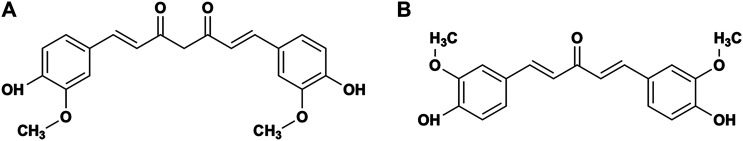
Molecular structure of **(A)** Curcumin and **(B)** 1,5-Bis (4-hydroxy-3-methoxyphenyl)-1,4-pentadien-3-one (MS13).

Curcumin (diferuloylmethane) (Figure 1A) is a naturally occurring polyphenolic compound extracted from *Curcuma longa* that demonstrated a variety of pharmacological activities, including anti-tumour, anti-inflammatory and chemopreventive properties (Masuda et al., 1993; Anand et al., 2008). Curcumin has been shown to exert anti-cancer effects against various human cancer cells, including pancreatic, lung, colorectal, breast and oral cancer cells, *in vitro* and *in vivo* (Duvoix et al., 2005; Costea et al., 2019). Cell-based and animal models studies have reported that curcumin induced cytotoxic cell death in both androgen-dependent PCa (ADPC) and AIPC (Dorai et al., 2001; Li et al., 2007; Teiten et al., 2010). Curcumin delays an early onset of PCa and inhibits the disease progression by modulating multiple key signalling pathways, including AR, AP-1, PI3K/Akt/mTOR, Wnt/ß-catenin, and several molecular targets such as NF-kB, Bcl-2 and cyclin D1 (Wahab et al., 2020). Also, curcumin has shown to selectively target malignant prostate cells compared to normal human prostate epithelial cells (Srivastava et al., 2007). Despite being categorized as Generally Recognised As Safe (GRAS) by the U.S. Food and Drug Administration (USFDA) (Lao et al., 2006), its applications in chemotherapeutic applications remain restricted due to low aqueous solubility, poor bioavailability and instability (Kudo et al., 2011; Aggarwal et al., 2014). These limitations prompted researchers to synthesize and design various analogues by modifying the curcumin structure to improve chemical stability and anti-cancer potency (Tomeh et al., 2019).

Diarylpentanoids (DAPs) have gained interest to researchers due to the high degree of anti-cancer activity and stability *in vitro* over its parent compound and other diarylheptanoids in a wide range of cancerous cells (Adams et al., 2004; Ohori et al., 2006; Liang et al., 2009; Katsori et al., 2011; Paulraj et al., 2019). These analogues comprise two aryl rings linked by a five-carbon spacer with deleted reactive ß-diketone moiety exhibited enhanced growth-suppressive activities against several cancer cells (Padhye et al., 2010; Qudjani et al., 2016; Moreira et al., 2020) and improved pharmacokinetics profiles (Vyas et al., 2013). DAPs have been shown to exhibit growth-suppressive activity and prevent tumour progression by modulating a variety of molecular targets and signalling pathways related to inflammation (STAT3, NF-kB), tumour invasion (VEGF), cell proliferation (MAPK/ERK, PI3K/Akt/mTOR, PTEN), cell cycle arrest and cell death (Bax, Bcl-2 and Bcl-xL, cleavage of caspase-2 and PARP) in both *in vitro and in vivo* models (Ohori et al., 2006). Studies of these monocarbonyl analogues that exhibited anti-cancer activity in various cancer cells, including PCa, has been published (Paulraj et al., 2019). Several studies have suggested that several DAPs, including RL118 (Chen et al., 2018), WZ35 (Zhang et al., 2015), Ca27 (Fajardo et al., 2012), Ca37 (Luo et al., 2014), EF24 (Yang et al., 2013) and MS17 (Citalingam et al., 2015) have potential as anti-cancer candidates for the treatment of PCa.

Previously, our group has reported that MS13 (1,5-Bis (4-hydroxy-3-methoxyphenyl)-1,4-pentadien-3-one), a DAP, (Figure 1B demonstrated growth inhibitory effects in time- and dose-dependent manner in prostate (Citalingam et al., 2015), cervical (Paulraj et al., 2015), glioblastoma, neuroblastoma (Lee et al., 2021), and colorectal (Ismail et al., 2020) cancer cell lines. Furthermore, MS13 has also shown to be effective than curcumin in inducing apoptosis by modulating apoptosis pathways in gastric (Yoshida et al., 2018), colorectal (Cen et al., 2009) and pancreatic (Friedman et al., 2009) cancer cells. Nonetheless, the anti-cancer effects and the gene expression profiling of MS13-treated AIPC cells have not been extensively studied. Hence in this present study, we evaluated the cytotoxic activity, anti-proliferative effect, apoptosis induction and anti-migration properties of MS13-treated DU 145 and PC-3 cells. In addition, we also investigated the underlying molecular mechanism of MS13 associated with cell cycle-apoptosis and PI3K pathways, the topmost significant targeted pathways by this compound. This study used two human PCa cell lines, DU 145 and PC-3 cells, which do not express androgen receptor (AR) mRNA and protein expression at the transcriptional level due to epigenetic modification of AR.

## Materials and Methods

### Preparation of Curcumin Analogue (MS13)

Curcumin analogue, MS13 (1,5-Bis (4-hydroxy-3-methoxyphenyl)-1,4-pentadien-3-one), with 326 molecular weight, is a chemically purified diarylpentanoid synthesized by coupling the appropriate aromatic aldehyde with acetone under base catalysed aldol condensation, using a 1:2 ratio of ketone to aldehyde. The compound was determined based on the analysis of spectroscopic data and the comparison of the data with the related compounds. Curcumin was purchased from Sigma Aldrich (Sigma-Aldrich, United States). The stock solution of both compounds was prepared by dissolving in dimethyl sulfoxide (DMSO, Molecular Biology Grade, Sigma-Aldrich, United States) to a final concentration of 50 mM.

### Maintenance of DU 145, PC-3 and WRL 68 Cells

The androgen-independent human prostate cancer cell lines (DU 145, ATCC^®^ HTB-81^™^ and PC-3, ATCC^®^ CRL-1435^™^) cells and human epithelial hepatocytes cell line (WRL-68, ATCC^®^ CL-48^™^) cells were purchased from American Type Culture Collection (ATCC, United States). DU 145 and WRL 68 cells were cultured in Eagle’s Minimum Essential Medium (EMEM) meanwhile, PC-3 cells were cultured in Ham’s F-12 medium (F-12K) (Mediatech, United States). These cells were incubated at 37°C in a humidified atmosphere of 5% CO_2_. All the cell lines were maintained in the respective media supplemented with supplemented with 10% (v/v) fetal bovine serum (FBS) (Gibco, United States) and 1% penicillin (100 U/mL)/streptomycin (100 μg/ml) (Gibco, United States).

### Cytotoxicity Analysis and Anti-proliferative Assay of MS13-Treated DU 145 and PC-3 Cells

Cells were plated in a 96-well plate culture plate (Nunc, Denmark) in triplicates at a density of 1 × 10^5^ cells/ml in 100 μL of culture media. The plate was incubated in a 5% CO_2_ incubator overnight at 37°C to allow cells attachments to the bottom of the wells. After 24 h, the media was replaced with fresh media containing MS13 or curcumin ranging from 1.56–100 μm and incubated at 37°C in a humidified incubator with 5% CO_2_. The cells were incubated for 72 h for cytotoxicity analysis and 24, 48 and 72 h for anti-proliferative assay. The sample compound was prepared in-stock solution of 50 mM in DMSO (Sigma-Aldrich, United States) and diluted in the culture media to working concentration 200 μM. Curcumin was used as a positive control was also prepared in a similar manner. A two-fold serial dilution was performed for each sample in triplicates down the columns of the plate, yielding a final volume and concentration of 100 μL in 0.2% DMSO in each well. Control cells were treated with media containing 0.2% of DMSO. The cell viability and anti-proliferative assay were determined by using 3-(4,5-dimethylthiazol-2-yl)-2,5-diphenyltetrazolium bromide (MTT) assay (Mosmann, 1983). Following incubation, the supernatant was aspirated, and 0.5 mg/ml MTT solution was added and incubated for 4 h at 37°C. To dissolve the formazan crystal, the MTT solution was replaced with 100 μL of DMSO. The absorbance was measured by Biotek EON (Biotek Instrument, United States) microplate reader at a wavelength 570/650 nm. The equation for cell viability percentage as follows:

$$\text{Cell}\;\text{viability}\;\left(\%\right)=\frac{\text{Absorbance}\;\text{of}\;\text{treated}\;\text{sample}\times 100}{\text{Absorbance}\;\text{of}\;\text{control}\;\text{sample}}$$

The half-maximal effective concentration (EC_50_) value was generated using GraphPad Prism version 7.0 (GraphPad Software, United States). EC_50_ value represents the concentration at which the tested compound caused 50% growth inhibitory effect averaged from triplicates absorbance values. Selectivity index (SX) is the degree of selectivity of the compound tested against the cancerous cell, in which values larger than “100” indicates the compound is selective towards cancerous cells and conders minimal toxicity toward non-cancerous cells (Popiołkiewicz et al., 2005; Chew et al., 2012). The value was calculated based on the equation below:

$$\text{Selectivity}\;\text{index}\left(\text{SX}\right)=\frac{{\text{EC}}_{50}\;\text{of}\;\text{cancer}\;\text{cell}\;\text{line}}{{\text{EC}}_{50}\;\text{of}\;\text{cancer}\;\text{cell}\;\text{line}}$$

### Morphological Observation of Apoptotic Cells by Acridine Orange/Propidium Iodide Double Staining Technique Using Fluorescence Microscope

The morphological assessment of cell death was performed using acridine orange (AO) and propidium iodide (PI) double staining. This method was used to differentiate between viable, non-viable cells and cells that undergo early or late apoptosis and necrosis. Cells were seeded in T25 cm^2^ flasks (Nunc) and incubated overnight until they reached 80–90% confluency. Cells were exposed to two different concentrations of MS13 for 24, 48 and 72 h, and each experiment included a set of control cells. A fresh clean pellet of the harvested cells was re-suspended in 150 ml ice-cold 1 × PBS. A mixture of the fluorescent dye staining solution (1:1), comprising 50 μg/ml Acridine Orange and 50 μg/ml Propidium iodide were prepared. A total of 10 µL of AO and PI were added to the pellet and incubated at room temperature in the dark for 5 min. The freshly stained cell suspension was dropped onto a glass slide and observed under a UV-fluorescence microscope (Olympus BX41) attached with Leica LAS X software. Fluorescent detection was observed under the fluorescence microscope using a dual filter set for FITC (green) and rhodamine (red). The different morphological criteria were used to clarify healthy live cells, early or late apoptotic and necrotic cells. The viable cells show an intact green nucleus with round intact structure, an early apoptosis display a dense bright green fluorescent cells exhibits distinct morphological changes like membrane blebbing, shrinkage of the cells and chromatin condensation. On the other hand, late apoptotic cells and necrotic cells will stain with both AO and PI. Hence, late apoptosis exhibits a yellowish orange to bright red cells with bright yellow beads nucleus whilst necrosis appeared as uniformly stained red with intact nuclei (Ng et al., 2013; Nordin et al., 2016). The percentage of viable, apoptotic, and necrotic cells were quantified by counting a minimum of 200 total cells was counted per sample, and the percentage of cells from each population was calculated according to the equation:

$$\text{Percentage}\;\text{of}\;\text{cells}=\frac{\text{number}\;\text{of}\;\text{viable}\;\text{or}\;\text{apoptotic}\;\text{or}\;\text{necrotic}\;\text{cells}\times 100}{200\;\text{cells}}$$

### Quantification of Caspase-3 Activity on MS13-Treated DU 145 and PC-3 Cells

Caspase-3 activity assay was performed using the Caspase-3 Colorimetric Assay Kit (Raybiotech Inc. GA, United States), following the manufacturer’s instruction. This assay is based on spectrophotometric detection of the chromophore p-nitroaniline (pNA) after cleavage from the labelled substrate DEVD-pNA, the pNA light emission can be quantified using a spectrophotometer at 400 or 450 nm. Firstly, cells were seeded in T75 cm^2^ flasks (Nunc) and then treated with MS13 at different concentration (EC_50_ and 2x EC_50_) at various time point 24, 48 and 72 h. Each experiment included a set of control cells (media with DMSO). Harvested cells were resuspended in chilled Cell Lysis Buffer, and the concentration of the protein lysate was determined by using Pierce BCA Protein Assay Kit (Thermo Scientific, United States) based on manufacturer’s protocols. The 2x Reaction Buffer (containing 10 mM DTT) was added to each sample for each assay. The reaction was started by the addition of 5 μL of the 4 mM DEVD-pNA substrate incubated at 37°C for 2 h. The absorbance was measured at 400 nm using a microplate spectrophotometer (BioTek™ EON™ Microplate Spectrophotometers, Fisher Scientific, United States). The data was presented in the fold-change of absorbance from treated cells against absorbance from control based on the equation below:

$$\text{Fold}\text{−}\text{change}=\frac{\text{Absorbance}\;\text{reading}\left(400\;\text{nm}\right)\text{of}\;\text{treated}\;\text{cells}}{\text{Absorbance}\;\text{reading}\left(400\;\text{nm}\right)\text{of}\;\text{untreated}\;\text{cells}}$$

### Quantification of Bcl-2 Cellular Protein Concentration Using Enzyme-Linked Immunosorbent Assay on MS13-Treated DU 145 and PC-3 Cells

Bcl-2 cellular protein concentration was quantified using the Human Bcl-2 Platinum ELISA Kit (Affymetrix eBioscience, Vienna, Austria) based on the manufacturer’s instruction. The kit is able to detect the human Bcl-2 by solid-phase sandwich ELISA which is designed to measure the amount of target bound between a matched antibody pair. The cells were seeded in T75 cm^2^ flasks (Nunc) and treated with different concentration of MS13 (EC_50_ and 2x EC_50_) at 24, 48 and 72 h. Each experiment included a set of control cells (media with DMSO). The concentration of protein lysate was also determined by using Pierce BCA Protein Assay Kit (Thermo Scientific, United States). The Bcl-2 concentrations of the samples were obtained by comparing the absorbance obtained against the standards at 450 nm by using a microplate spectrophotometer (BioTek™ EON™ Microplate Spectrophotometers, Fisher Scientific, United States). Data was presented in the fold-change of absorbance from treated cells against absorbance from untreated cells (control) based on the equation below:

$$\text{Fold}-\text{change}=\frac{\text{Absorbance}\;\text{reading}\left(450\;\text{nm}\right)\text{of}\;\text{treated}\;\text{cells}}{\text{Absorbance}\;\text{reading}\left(450\;\text{nm}\right)\text{of}\;\text{untreated}\;\text{cells}}$$

### Cell Migration Inhibition Assay (In-Vitro Scratch-Wound Healing Assay) on MS13-Treated DU 145 and PC-3 Cells

Cells were seeded in triplicates in 24 well plates (Nest, Denmark) overnight at 37°C in a humidified incubator to a nearly confluent cell monolayer. The monolayer cells then carefully scratched using a yellow pipette tip to draw a liner “wound” of each well, and the debris was removed. The cells were treated with MS13 (EC_50_ and 2× EC_50_), incubated at 37°C, and photographed under a microscope for 0, 6, 12, 24, and 30 h. The control well was added with media and DMSO. The anti-migration ability of MS13 was determined by using *in vitro* scratch wound assay and analysed by using the ImageJ software based on the method described by Liang et al., 2007 (Liang et al., 2007). The area of the wounds was measured using an ImageJ software macro tool; MRI wound healing tool on Fiji (Fiji Is Just ImageJ) platform (Schindelin et al., 2015). The area measurements of each wound were converted into percentage by using the formula:

$$\begin{array}{l} \%\;\text{of}\;\text{scratch}\;\text{wound}\;\text{closure}:100\%-\%\;\text{of}\;\text{wound}\;\text{remaining}*\\ {}*\text{wound}\;\text{remaining}=\frac{\text{measurement}\;\text{at}\;\text{time}\left(6,12,24,30,48\;\text{h}\right)\times 100}{\text{measurement}\;\text{at}\;\text{time}\;0\;\text{hour}} \end{array}$$

### Statistical Analysis

All samples were measured in triplicates from three independent experiments. Comparison between sets of data was performed by using one way and two-way analysis of variance (ANOVA) followed by Dunnett’s multiple group comparison test. Results presented as mean ± standard deviation (SD). Statistically significant differences between groups were accepted at *p* ≤ 0.05. All statistical analyses were performed using GraphPad Prism Version 7.0 (GraphPad Software Inc., La Jolla, CA, United States).

### Induction of Apoptosis of MS13-Treated Cell Lines

In order to examine the gene expression profiling affected by MS13 on the prostate cancer cell lines, both DU 145 and PC-3 cells were seeded at the density of 1 × 10^7^ cells in T75 cm^2^ flasks (Nunc) and incubated overnight to allow cells attachment at 37 °C in a humidified incubator with 5% CO_2_. The cells were incubated with 16 μM of MS13 for 24 h duration based on the EC_50_ values. Meanwhile, the untreated cells received only media with only DMSO. After 24 h treatment, the treated cells were harvested by pre-warmed accutase® Cell Detachment Solution (Innovative Cell Technologies, San Diego, CA, United States), washed with 2 ml of 1× PBS (Corning^®^) and centrifuged twice to obtain a fresh clean pellet. Then, the fresh pellet was re-suspended in 1,000 ml of 1x PBS (Corning^®^), kept on ice and further to RNA extraction. The experiment was performed in three biological replicates.

### Total RNA Extraction and Gene Expression Analysis

Total RNA was extracted from MS13-treated DU 145 and PC-3 cells and untreated controls using the RNeasy Mini Kit (Qiagen, Valencia, CA, United States) as per the manufacturer’s instructions. The QIAshredder spin columns (Qiagen, Valencia, CA, United States) was used for homogenization. RNA concentration and purity were determined by using a NanoPhotometer (Implen, Munich, Germany). A ratio of absorbance at 260/280 nm ∼2.0 was accepted as pure and was used for Nanostring^®^ analysis. A nCounter PanCancer Pathway Panel (Nanostring Technologies, Seattle, WA, United States) was used. Triplicate wells were assayed for each experiment, and three independent experiments were performed.

### Gene Expression Analysis

Gene expression profiling was performed by Nanostring^®^ nCounter Technology (Nanostring Technologies, Seattle, WA, United States), using the nCounter PanCancer Pathway Panel (Nanostring Technologies, Seattle, WA, United States), which consists of a panel 770 genes associated with 13 pathways of cancer. Results were analysed using the Nanostring nSolver 4.0 and Advanced Analysis Module, plugin (Ver.2) with R statistical software and normalization of housekeeping genes were obtained from the geNorm algorithm. Differential expression of the treated cells was compared with untreated cells (control), with the fold change and *p* values calculated using nSolver default settings. Genes were considered as significant differentially expressed genes (DEGs) based on the criteria of fold-change (FC) ≥ 2, *p*. adjusted ≤0.05, and a false discovery rate (FDR) ≤ 0.05.

## Results

### Cytotoxicity and Anti-proliferative Effects of MS13-Treated DU 145 and PC-3 Cells

**FIGURE 2:**
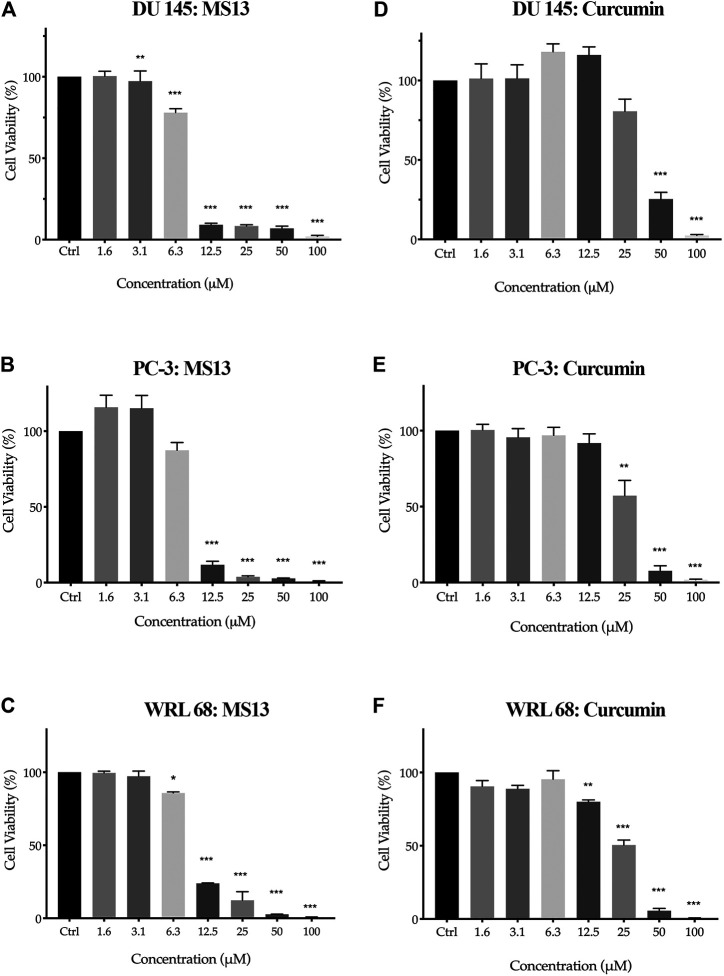
The cell viability effects of MS13 and curcumin against DU 145, PC-3 and WRL 68 compared to control **(A)** DU 145 for MS13, **(B)** PC-3 for MS13, **(C)** WRL 68 for MS13, **(D)** DU 145 for Curcumin, **(E)** PC-3 for Curcumin and **(F)** WRL 68 for Curcumin. Experiments were performed in triplicates, and results are compared between three independent experiments (*n* = 3) by using ANOVA. Results are expressed as means ± SE. *p ≤ 0.05, **p ≤ 0.001, ***p ≤ 0.0001 indicates statistically significant differences between the means of values obtained with treated vs untreated cells.

To evaluate the *in vitro* dose-dependent cytotoxicity and selectivity index effects, DU 145, PC-3 and WRL 68 (normal human liver epithelia) cell lines were incubated with different concentration of MS13. Overall, MS13 induced cytotoxic effects against DU 145 and PC-3 cells in a dose-dependent manner. MS13-treated DU 145 cells has shown a significant reduction of cell viability beginning from 3.1 μM onwards to about 97%. The cell viability was reduced to 78% at 6.3 μM, followed by less than 10% from 12.5 to 100 μM. Meanwhile, MS13-treated PC-3 cells has shown a significant reduction of cell viability by approximately 88% at 12.5 μM and continue to decrease below than 4% from 25 to 100 μM. Besides, curcumin showed a significant cell inhibitory effect at 50 μM with 25% cell viability in DU 145. However, in PC-3 treated cells, 57% cell viability was noted at 25 μM, while 10% was observed at 50 μM (Figures 2A,B,D,E). Hence, this indicated that curcumin is less cytotoxic compared to MS13 against both cell lines.

MS13 has exhibited lower EC_50_ values (DU 145; 7.57 ± 0.2 μM and PC-3; 7.80 ± 0.7 μM) while curcumin displayed a higher value of EC_50_ (DU 145; 34.25 ± 2.7 μM and PC-3; 27.77 ± 6.4 μM). Furthermore, MS13 also exhibited lower EC_50_ value for WRL 68 cells (9.31 ± 0.2 μM) compared to curcumin (26.45 ± 3.3 μM). (Table 1) this indicated that a higher dose of MS13 was required to decrease the cell viability of WRL 68 by 50% compared to PCa cells.

**TABLE 1:** The EC_50_ values of MS13 and curcumin on DU 145 and PC-3 and normal epithelial hepatocytes cell line (WRL 68) for 72 h incubation time.

Compounds	EC_50_ values (μM) of MS13 on cell lines		
	DU 145	PC-3	WRL 68
MS13	7.57 ± 0.2	7.80 ± 0.7	9.31 ± 0.2
Curcumin	34.25 ± 2.7	27.77 ± 6.4	26.45 ± 3.3

^a^Curcumin was used as a positive control. Results are shown as a mean ± standard error from three independent experiments.

Selective index values of MS13 and curcumin were also evaluated where the value greater than 100 suggests that the compounds are less toxic to normal cells but greater toxicity towards PCa cells. MS13 showed higher SX values in both PCa cells (DU 145; 122.8 μM and PC-3; 119.17 μM) compared to curcumin (DU 145; 77.2 μM and PC-3; 95.25 μM)(Table 2). In summary, MS13 treatment on DU 145 and PC-3 cells showed a higher potency with a lower dose-dependent cytotoxic effect compared to curcumin. MS13 also exhibits low toxicity against normal human cell lines compared to curcumin.

**TABLE 2:** The selective index (SX) values of MS13 and curcumin on each tested prostate cancer cell lines (DU 145 and PC-3) and normal epithelial hepatocytes cell lines (WRL 68) for 72 h incubation time.

Compounds	WRL68 (normal human epithelial hepatocytes)	
	DU 145 cells	PC-3 cells
MS13	122.8	119.17
Curcumin	77.2	95.25

SX values >100 indicate that the cytotoxicity effect of the tested compound is greater towards cancer cells.

**FIGURE 3:**
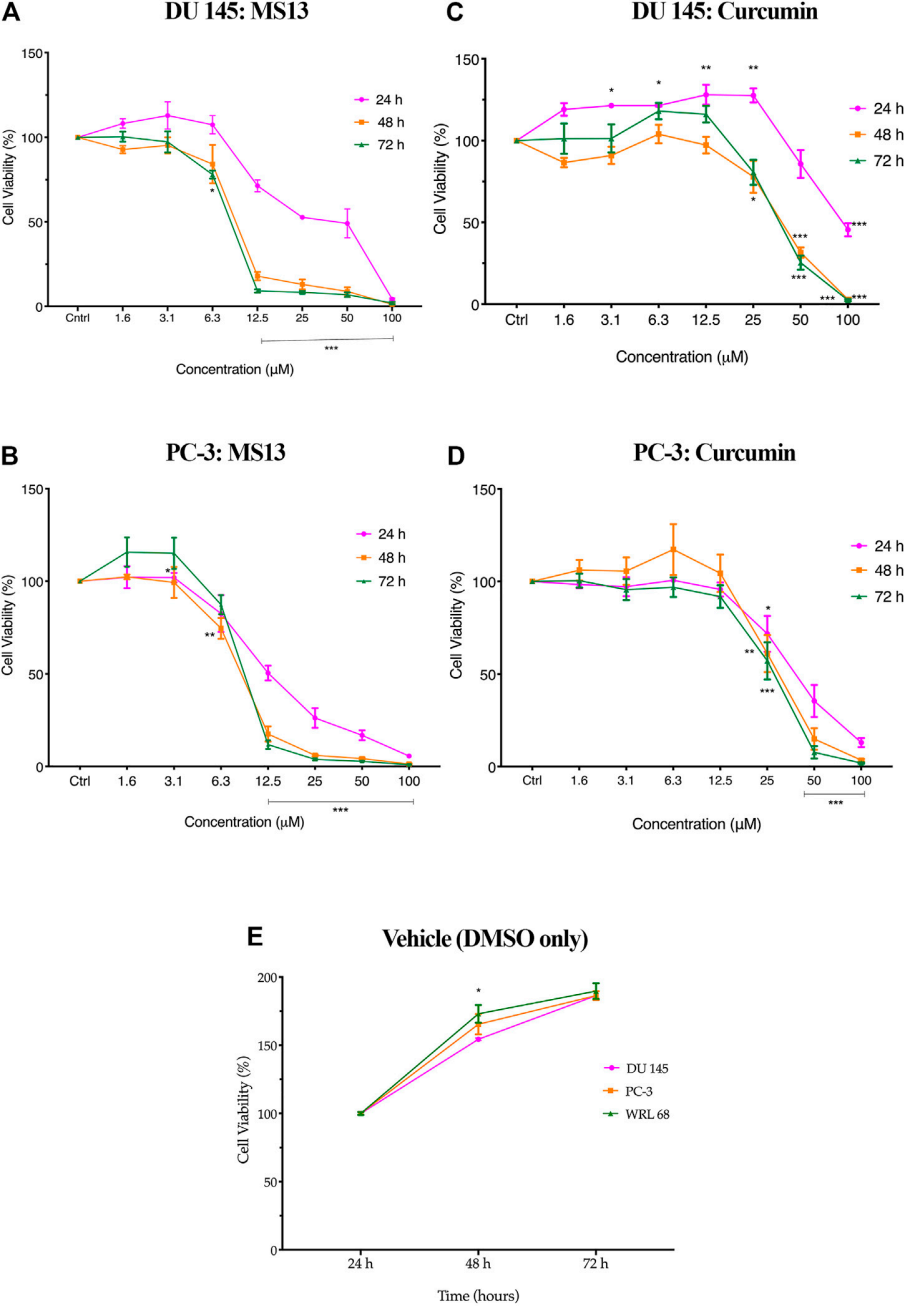
The anti-proliferation effect of MS13 and curcumin against DU 145 and PC-3 cell lines at 24, 48, and 72 h. Experiments were performed in triplicates, and results are compared between three independent experiments (*n* = 3) by using ANOVA. Results are expressed as means ± SE. *p ≤ 0.05, **p ≤ 0.001, ***p ≤ 0.0001 indicates statistically significant differences between the means of values obtained with treated vs untreated cells (control).

The evaluation of the anti-proliferative activity has shown that MS13 inhibited the proliferation of DU 145 and PC-3 cells in a dose- and time-dependent manner. The percentages of cell viability of the cells were decreased as the concentration of MS13 increased and prolonged exposure time. MS13-treated DU 145 cells have displayed significant inhibition of cell proliferation at 12.5 μM onwards for 24, 48 and 72 h. At 24 h, the cell viability was significantly reduced by approximately 29% and about 66 and 91% at 48 and 72 h, respectively. A significant gradual reduction of cell proliferation was observed when treated with 25–100 μM by 47–98% for all time points (Figure 3A). Likewise, a significant reduction of anti-proliferative activity in MS13-treated PC-3 cells was observed from 12.5 μM onwards by approximately 50% at 24 h, and 82 and 88% at 48 and 72 h, respectively. Doses from 25 to 100 μM were continuously showed a significant reduction of cell viability by 75–99% for all time points (Figure 3B). As for curcumin, the treatment on DU 145 cells displayed a significant reduction in cell proliferation only at 100 μM at 24 h and 50 μM at both 48 and 72 h. On the other hand, curcumin treatment on PC-3 cells showed a significant reduction at 50 μM for 24 and 48 h while at 25 μM for 72 h (Figure 3C,D). It was also noted that the vehicle-treated controls (DMSO only) have shown a significant increase in the cell viability between 24 and 72 h, indicating that cell proliferation increases as a function of time. But a decrease in cell viability was noted in cells treated with MS13 (Figure 3E). As a summary, these results indicated that MS13 exhibited low toxicity, enhanced cytotoxicity and greater anti-proliferative activity against DU 145 and PC-3 cells compared to curcumin.

### Morphological Assessment of Apoptotic Cells by Acridine Orange/Propidium Iodide Double Staining Technique Using Fluorescence Microscope

**FIGURE 4:**
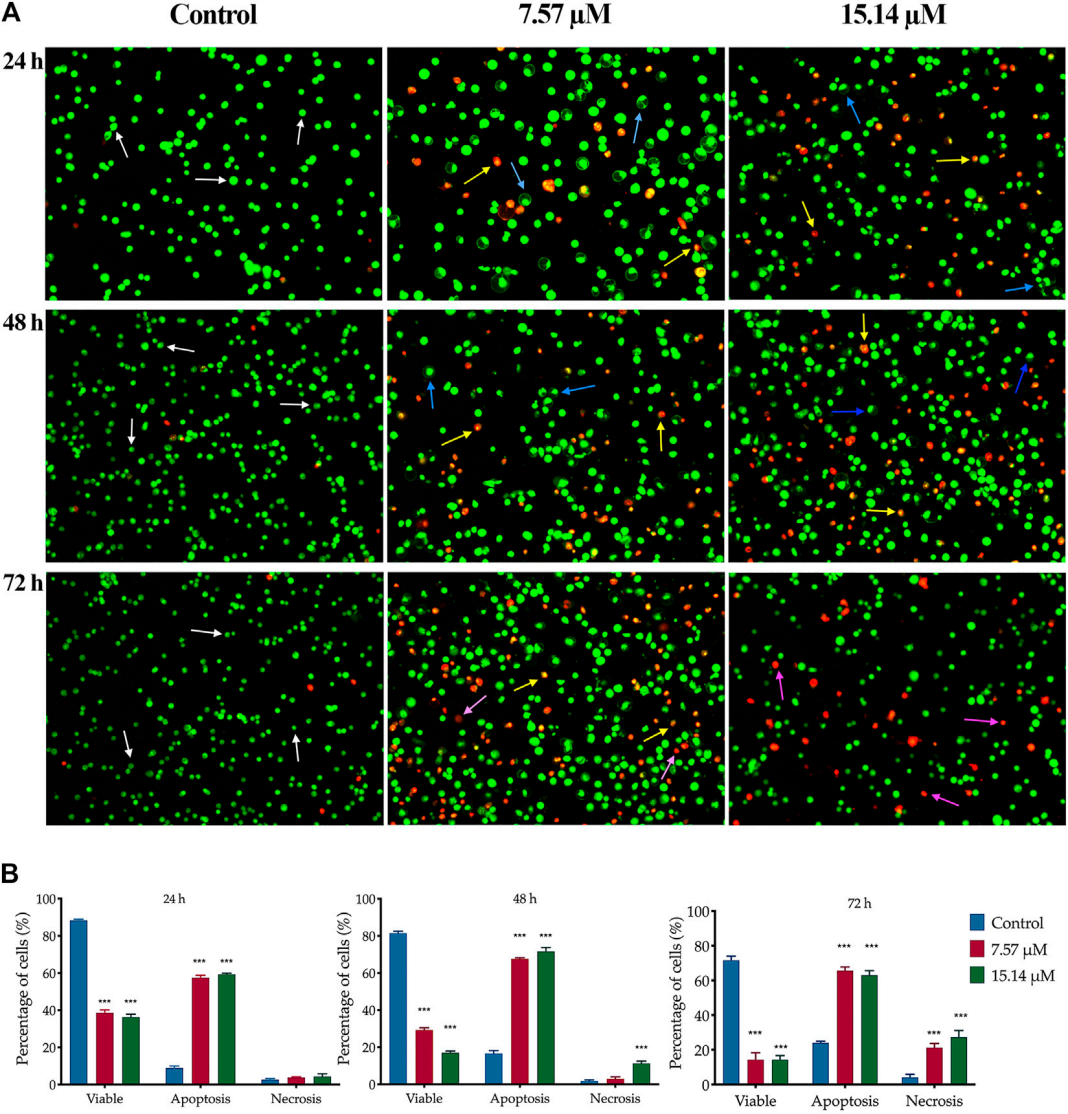
Morphological and quantitative analysis of apoptotic cells by acridine orange/propidium iodide (AO/PI) in MS13-treated DU 145 cells. **(A)** Detection by fluorescent microscopy of AO/PI double-stained DU 145 cells treated with MS13 at 7.57 and 15.14 µM for 24, 48 and 72 h. Untreated viable cells emit uniformly green fluorescence (white arrow), while early apoptotic cells emit a dense bright green fluorescence, exhibited membrane blebbing and chromatin condensation (blue arrow). Late apoptosis cells stained with a dense bright orange-red with visible yellow beads (yellow arrow). Necrotic cells showed a red appearance (purple arrow). Magnification 100×. h–hours. **(B)** Percentage of cell populations in DU 145 treated with MS13 for 24, 48, and 72 h. Treated and non-treated cells were double-stained with AO/PI. Minimum 200 cells were counted per sample, and the percentage of cells from each population (viable, apoptosis and necrosis) was calculated. Results are expressed as means ± SE. Experiments were performed in triplicates, and results are compared between three independent experiments (*n* = 2) by using ANOVA. *p ≤ 0.05, **p ≤ 0.001, ***p ≤ 0.0001 indicates statistically significant differences between the means of values obtained with treated vs untreated cells (control).

The apoptotic changes in terms of colour and morphology in MS13-induced DU 145 and PC-3 cells were analysed at 24, 48 and 72 h by using acridine orange (AO) and propidium iodide (PI) double staining technique. In MS13-treated DU 145, under untreated condition, viable cells are characterized by intact green-coloured cells exhibited a spherical structure, indicating no signs of apoptosis. After 24 h of incubation with 7.57 µM of MS13, a dense bright green fluorescent cells exhibited distinct morphological changes like membrane blebbing, shrinkage of the cells and chromatin condensation were increased, signifying a shift from viable to early apoptotic cells. As the concentration of MS13 increased to 15.14 μM, a higher proportion of a yellowish orange to bright red cells were observed, indicating the majority of the cells underwent late apoptosis. Meanwhile, after 48 h of incubation with 7.57 μM, a heterogeneous population of early and late apoptotic cells was noted, exhibited by a higher intensity of a dense bright green and a yellowish orange to bright red fluorescence cells. This indicated an increasing number of apoptotic cells with an increasing length of incubation. An increased number of cells turned from a dense yellowish orange to bright red cells was observed as the concentration of MS13 increased. Upon 72 h of incubation, as the mixture of cells emits dense yellowish orange to bright red cells and distinct apoptotic bodies increased, a proportion of reddish cells that defined necrotic cells were also increased. A higher proportion of uniform red or orange-stained cells were observed as the MS13 dose was increased (Figure 4A).

**FIGURE 5:**
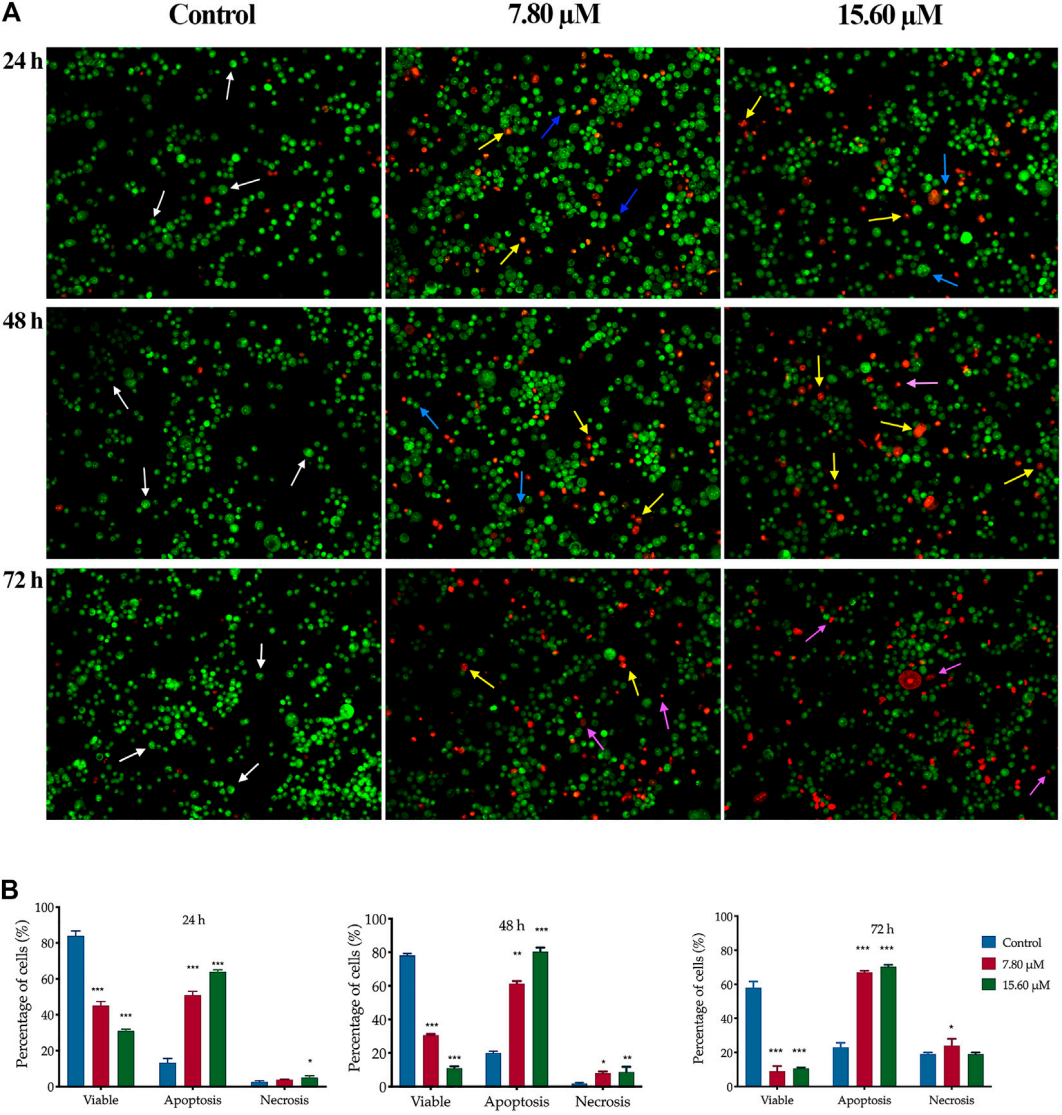
Morphological and quantitative analysis of apoptotic cells by acridine orange/propidium iodide (AO/PI) in MS13-treated PC-3 cells. **(A)** Detection by fluorescent microscopy of AO/PI double-stained PC-3 cells treated with MS13 at 7.80 and 15.60 µM for 24, 48 and 72 h. Untreated viable cells emit uniformly green fluorescence (white arrow), while early apoptotic cells emit dense bright green fluorescence exhibited membrane blebbing and chromatin condensation (blue arrow). Late apoptosis cells stained with dense bright orange-red with visible yellow beads (yellow arrow). Necrotic cells showed a red appearance (purple arrow). Magnification 100 x. h–hours. **(B)** Percentage of cell populations in PC-3 cells treated with MS13 for 24, 48, and 72 h. Treated and non-treated cells were double-stained with AO/PI. Minimum 200 cells were counted per sample, and the percentage of cells from each population (viable, apoptosis and necrosis) was calculated. Results are expressed as means ± SE. Experiments were performed in triplicates, and results are compared between three independent experiments (*n* = 2) by using ANOVA. *p ≤ 0.05, **p ≤ 0.001, ***p ≤ 0.0001 indicates statistically significant differences between the means of values obtained with treated vs untreated cells (control).

A similar pattern of cell proportion was observed in MS13-treated PC-3 cells (Figure 5A). At the treatment concentration of 7.80 µM after 24 h displayed a dense bright green-stained cells exhibited distinct morphology features of apoptosis and a dense bright orange-red stained cells indicating early and late apoptotic cells (Zimmermann et al., 2001). As the time prolonged to 48 and 72 h, a higher proportion of mixture between a dense bright green and a dense bright orange-red stained cells was seen. A dense bright orange-red cells increased after treatment with 15.60 µM, signifying abundant cells in the late stages of apoptosis. At 72 h, a number of cells stained red was observed when treated with 7.80 µM and become more apparent as the dose of MS13 increased. Nevertheless, the number of uniform red-stained cells was generally very low in both MS13-treated cells, suggesting that cell death occurred mainly due to apoptosis rather than necrosis. Overall, the morphological observation revealed that MS13 could induce apoptosis in DU 145 and PCa cells in a time and dose-dependent manner.

### Quantitative Analysis of the Morphology Assessment of Apoptotic Cells by Acridine Orange/Propidium Iodide (AO/PI) Double Staining Technique Using Fluorescence Microscope

The percentage of viable, apoptotic and necrotic cells were quantitatively determined. An early and late apoptosis groups were combined to indicate an apoptotic activity for this analysis. In MS13-treated DU 145 cells, the percentage of viable cells was significantly decreased at 24 h in a dose-dependent manner when treated with 7.57 μM (39%) and 15.14 μM (36%) compared to the control (66%). After 48 h incubation, there was a greater reduction in the cell viability to 29 and 17% for 7.57 and 15.14 μM, respectively. The percentage of viable cells was further reduced significantly to 14% for both doses after 72 h. Untreated cells exhibited a high percentage of viable cells, with 57 and 45% after 48 and 72 h. The percentage of cells that underwent apoptosis at 24 h was relatively low at all treatment doses; 7.57 μM (58%) and 15.14 μM (59%). However, the percentage of apoptotic cells increased after 48 h with 68 and 72% when treated with 7.57 and 15.14 μM, respectively. The untreated cells that underwent apoptosis were relatively low for 24 h (32%) and 48 h (41%), including after 72 h (50%). The percentage of the necrotic cells was relatively low at 24 h, approximately 4% for all doses and increased from 3 to 11% at 48 h. Upon 72 h, the percentage of apoptotic cells showed 66 and 63% for both doses at 72 h. Although apoptotic was relatively high at 72 h but, necrotic cells were noted to increase at this time up to approximately 20–30% for 7.57 and 15.14 μM, respectively (Figure 4B).

The same dose-dependent pattern was shown in MS13-treated PC-3 cells. A significantly higher apoptotic activity was shown at 48 h when treated with 7.80 μM (61%) and 15.60 μM (80%) with relatively low necrotic cells, 8 and 9%, respectively. At 72 h, the percentage of apoptotic cells increased when treated with 7.8 μM (67%), with a slight increment following treatment with 15.60 μM (70%). The necrotic cells were highest at 72 h compared to 24 and 48 h with 20% for 7.8 μM and 24% for 15.60 μM. Besides, the viable cells were decreased as the doses increased compared to the control (Figure 5B). In summary, MS13 induced higher apoptotic activity at 24 and 48 h with a low percentage of necrosis in both PC-3 and DU 145 cells in a time- and dose-dependent manner.

### Caspase-3 Activity and Bcl-2 Protein Concentration on MS13-Treated DU 145 and PC-3 Cells

**FIGURE 6:**
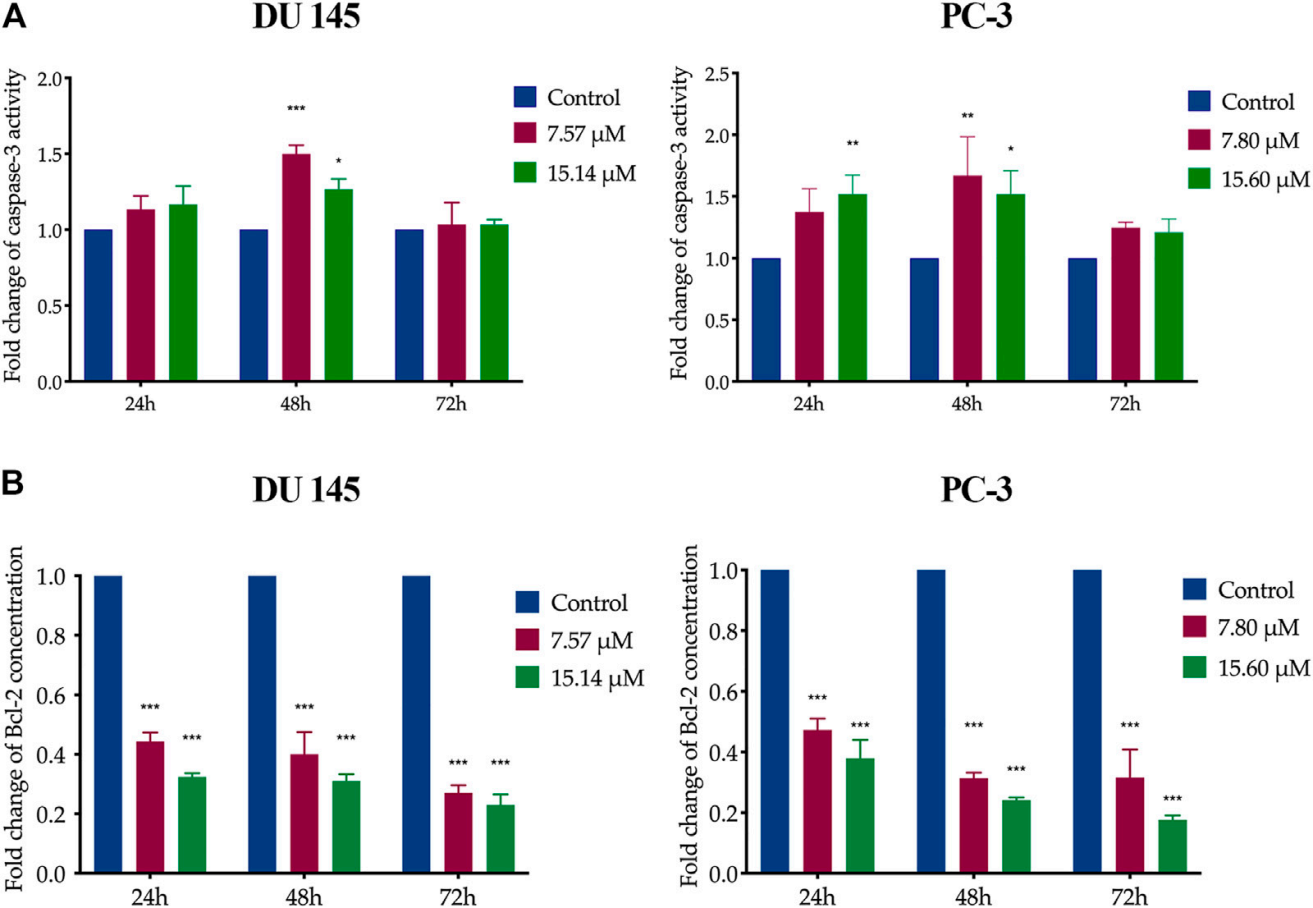
Caspase-3 activity and Bcl-2 protein concentration on MS13-treated DU 145 and PC-3 cells. **(A)** Caspase-3 activity in DU 145 and PC-3 cells measured at 24, 48 and 72 h. **(B)** Bcl-2 protein concentration in DU 145 and PC-3 cells treated with MS13 measured at 24, 48 and 72 h. Results are expressed as means ± SE. Experiments were performed in duplicates, and results were compared between two independent experiments (*n* = 2) using ANOVA. *p ≤ 0.05, **p ≤ 0.01, ***p ≤ 0.001 indicate statistically significant differences between the means of values obtained with treated vs untreated cells (control).

In MS13-treated DU 145 cells, the results showed a significant increase of caspase-3 activity at 48 h when treated with 7.57 μM. However, caspase-3 activity was slightly decreased as the dose increased to 15.14 μM. A similar trend was observed in MS13-treated PC-3 cells incubated with 7.80 and 15.60 μM at 48 h. There were no significant differences in caspase-3 activity at 72 h for both doses compared to untreated cells in both cells. In PC-3 cells, treatment with 7.80 μM showed no significant increase of caspase-3 activity at 24 and 72 h, but an increase was observed with 15.6 μM treatment for 24 h compared to untreated cells. The results indicated that the MS13 induces the highest caspase-3 activity at 48 h in both DU 145 and PC-3 cells (Figure 6A).

On the other hand, data analysis showed a significant and progressive decrease in Bcl-2 concentration from 24 to 72 h when treated with 7.57 and 7.80 μM of MS13 in DU 145 and PC-3 cells, respectively. However, increased doses of MS13 to 15.14 μM for DU 145 and 15.60 μM for PC-3 with a similar incubation period, a further reduction of Bcl-2 concentration was noted (Figure 6B). Hence, the results suggest that MS13 significantly suppress the Bcl-2 protein concentration in a dose and time-dependent manner for both PCa cells. Therefore, MS13 has shown the occurrence of apoptosis in a time and dose-dependent manner by increasing the caspase-3 activity while reducing the level of Bcl-2 protein treated in AIPC cells.

### Cell Migration Analysis (*In vitro* Scratch Wound Healing Assay) on MS13-Treated DU 145 and PC-3 Cells

**FIGURE 7:**
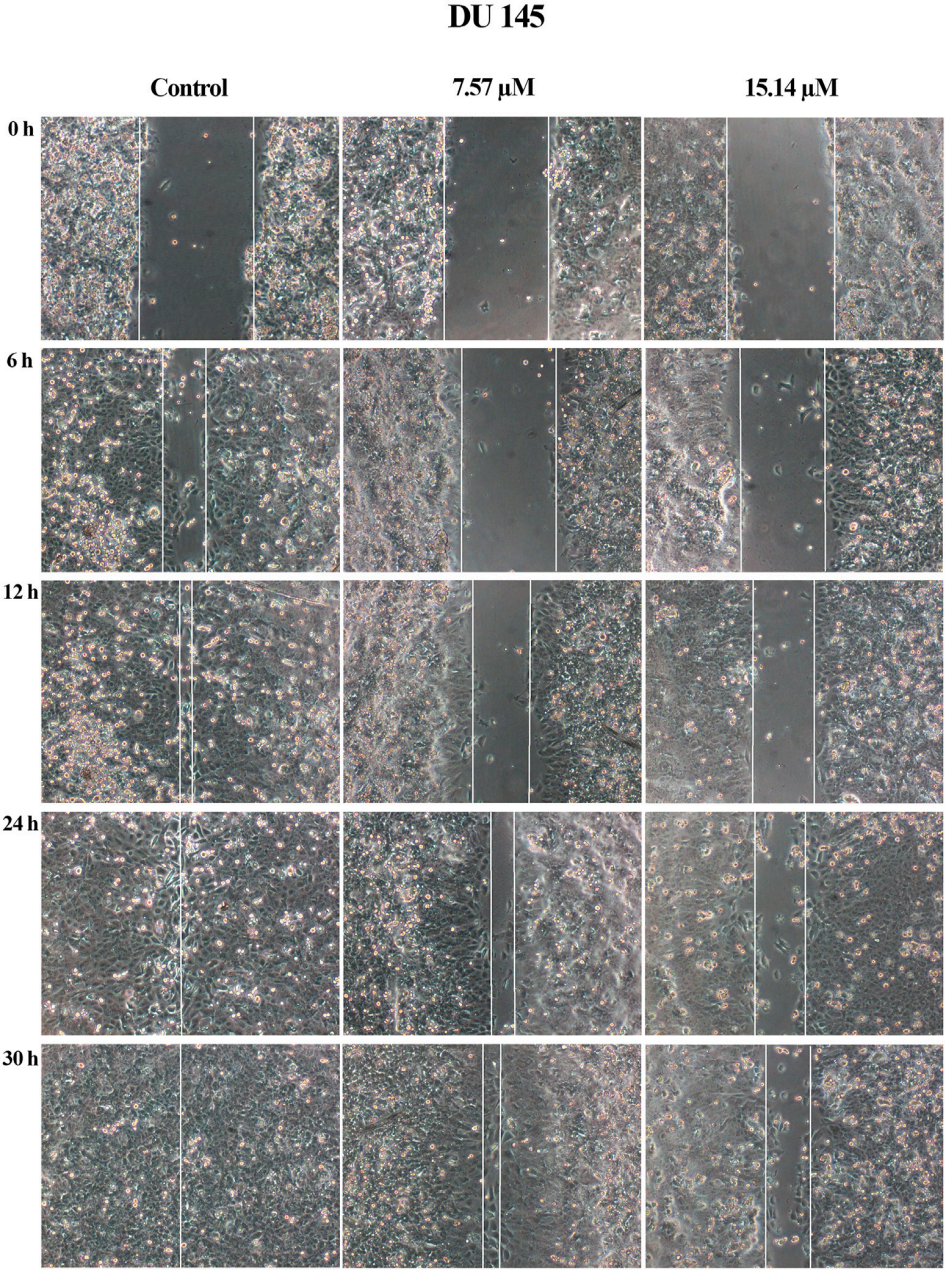
Cell migration photomicrographs from *in vitro* scratch wound healing assays of DU 145 cells treated with MS13 at 7.57 and 15.14 μM. The controls were treated with media and DMSO. The same area was captured at 0, 6, 12, 24 and 30 h after wound infliction. The cell-free region is showed with the white line.

**FIGURE 8:**
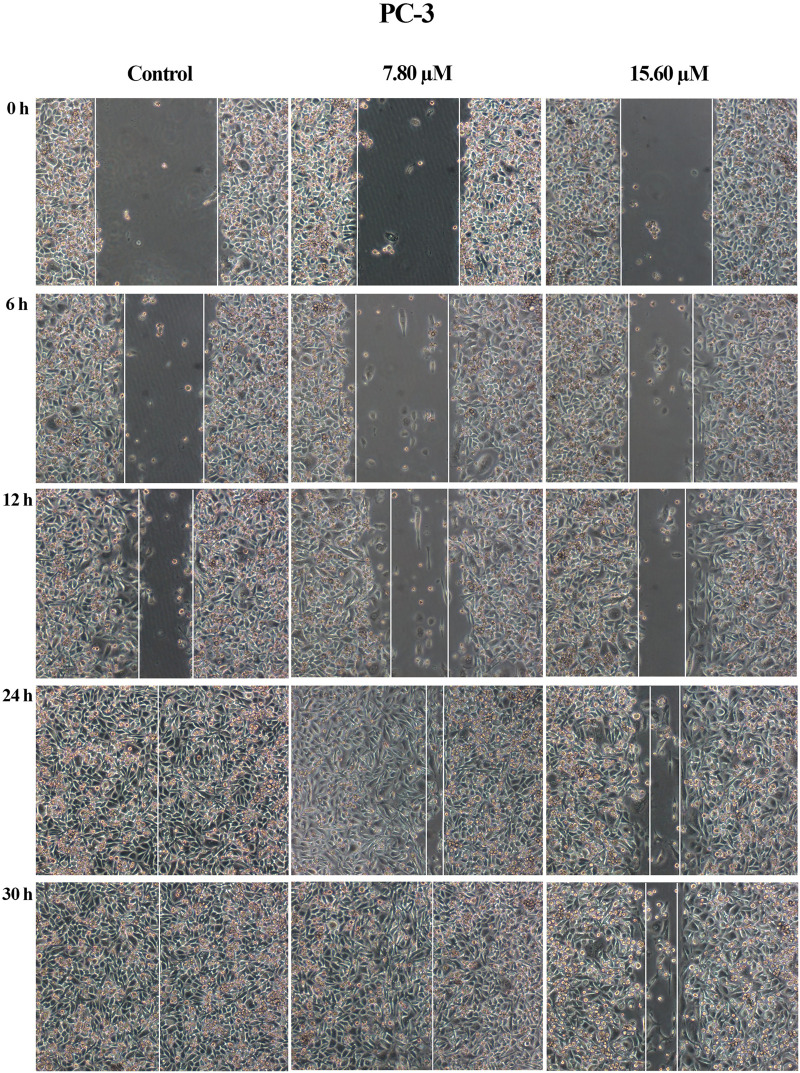
Cell migration photomicrographs from *in vitro* scratch wound healing assays of PC-3 cells treated with MS13 7.80 and 15.60 μM. The controls were treated with media and DMSO. The images from the same area were captured at 0, 6, 12, 24 and 30 h after wound infliction. The cell-free region is showed with the white lines.

**FIGURE 9:**
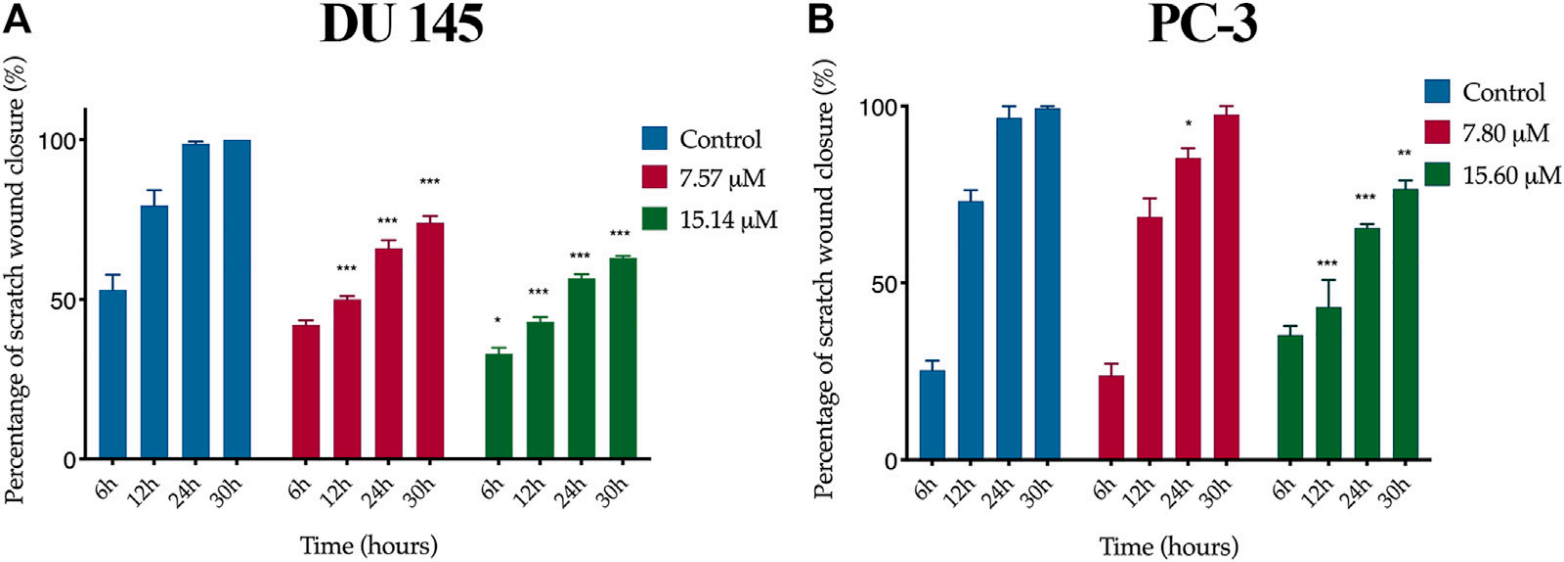
Quantification of cell migration of **(A)** DU 145 and **(B)** PC-3 cells treated with MS13. Controls were treated with media and DMSO only. The wound width was measured at 0, 6, 12, 24 and 30 h. The free-region cells after scratch were compared with the area of 0 h. Results are expressed as means ± SE. Experiments were performed in triplicates, and results are compared between three independent experiments (*n* = 3) by using ANOVA. *p ≤ 0.05, **p ≤ 0.001, ***p ≤ 0.0001 indicates statistically significant differences between the means of values obtained with treated vs untreated cells (control).

A wound healing assay was performed to assess the effect of MS13 on cell migration. The results have shown that the control filled the entire wound gap after 24 h for DU 145 and PC-3 cells (Figure 7 and Figure 8). Quantitatively, the percentage of scratch wound closure for untreated cells at 24 and 30 h following wound infliction were from 97 to 100% for both cells (Figure 9). Overall, MS13 treatment reduced wound closure, indicating significant inhibition of cell migration in DU 145 and PC-3 cells, compared with control. MS13-treated DU 145 cells exhibited wider wound gaps even after 24 and 30 h of treatment with 7.57 and 15.14 μM. Treatment with 7.57 μM MS13 have shown a significant decrease in the percentage of wound closure area with 50–74% from 12 to 30 h. As the dose increased (15.14 μM), the results showed a greater reduction of wound closure percentage ranging from 33 to 63% for all time points, indicating the anti-migratory role of MS13 (Figure 7 and Figure 9A). In contrast, PC-3 cells treated with 7.80 μM MS13 has shown a completely covered wound at 30 h. Data has shown that the wound closure was not significantly decreased when incubated with 7.80 μM compared to control for 6, 12 and 30 h but significant at 24 h time point. Meanwhile, treatment with 15.60 μM has shown wider gaps remained at 30 h. There was a significant decrease of wound closure percentage at 43, 66 and 77% for 12, 24 and 30 h, respectively in 15.60 μM MS13-treated PC-3 cells (Figure 8 and Figure 9B). The findings indicated the anti-migratory role of MS13 in DU 145 and PC-3 treated cells.

### Gene Expression of MS13-Treated DU 145 and PC-3 Cells

Anti-cancer properties of MS13 on DU 145 and PC-3 was further investigated using nCounter PanCancer Pathway Panel (Nanostring Technologies, United States) gene expression analysis. Based on the apoptosis induction studies, the doses of MS13 at 16 µM (2x EC_50_) were used for both DU 145 and PC-3 cells, incubated for 24 h. Genes with a filter of two-fold-cut-off, *p*. adjusted ≤0.05, and FDR ≤0.05 were defined as significant differentially expressed genes (DEGs). Overall, the data revealed that MS13 affected mutually regulated DEGs which associated with 12 canonical cancer pathways, including MAPK, JAK-STAT, PI3K, RAS, cell cycle-apoptosis, Wnt, DNA damage repair, driver gene, transcriptional misregulation, chromatin modification, notch and TGF-ß pathways.

However, our findings indicated that the cell cycle-apoptosis and PI3K pathways were the most significantly dysregulated following MS13 treatment. A total of 18 mutually DEGs was associated with cell cycle-apoptosis pathway, of which 14 were downregulated, and 4 were upregulated (Table 3). Besides, MS13 treatment has also altered the expression of 7 DEGs associated with PI3K pathway, 4 were downregulated, and 3 were upregulated (Table 4). Therefore, the mutually regulated DEGs associated with cell–cycle apoptosis and PI3K pathways were selected for further analysis that may implicate MS13 anti-cancer activity.

**TABLE 3:** Mutually regulated differentially expressed genes (DEGs) associated with cell cycle-apoptosis pathway following 16 μM treatment of MS13 in DU 145 and PC-3 cells at 24 h.

Gene symbol	Gene name	Accession	DU 145		PC-3	
			Fold-change	*p*-value^a^	Fold-change	*p*-value
AKT1	v-akt murine thymoma viral oncogene homolog 1	NM_005163.2	−2.5	6.54E-07	−2.0	1.71E-06
CCNA2	Cyclin A2	NM_001237.2	−4.9	6.57E-09	−2.8	8.74E-06
CCNB1	Cyclin B1	NM_031966.2	−7.0	9.93E-09	−2.3	5.56E-06
CDC25B	Cell division cycle 25B	NM_021873.2	−4.9	6.41E-06	−2.8	8.59E-07
CDC25C	Cell division cycle 25C	NM_001790.2	−2.5	5.09E-04	−2.2	0.000159
CDC7	Cell division cycle 7	NM_003503.2	−2.9	9.88E-06	−2.8	0.000244
CDKN1A	Cyclin-dependent kinase inhibitor 1A (p21, Cip1)	NM_000389.2	5.9	3.97E-08	7.6	4.21E-06
ENDOG	Endonuclease G	NM_004435.2	−3.2	1.24E-05	−2.3	0.000182
GADD45A	Growth arrest and DNA-damage-inducible, alpha	NM_001924.2	10.0	2.73E-09	11.6	3.4E-08
GADD45B	Growth arrest and DNA-damage-inducible, beta	NM_015675.2	2.5	2.17E-05	16.8	2.12E-06
PIK3R3	Phosphoinositide-3-kinase, regulatory subunit 3 (gamma)	NM_003629.3	2.3	0.000139	2.5	9.32E-05
PKMYT1	Protein kinase, membrane associated tyrosine/threonine 1	NM_004203.3	−2.8	9.83E-04	−2.3	0.00296
PPP3CB	Protein phosphatase 3, catalytic subunit, beta isozyme	NM_001142354.1	−2.7	0.00112	−2.0	0.000859
PRKDC	Protein kinase, DNA-activated, catalytic polypeptide	NM_006904.6	−2.5	1.94E-07	−2.5	1.45E-05
SKP2	S-phase kinase-associated protein 2, E3 ubiquitin protein ligase	NM_005983.2	−2.6	3.26E-05	−2.0	0.000171
SMAD3	SMAD family member 3	NM_005902.3	−2.3	2.41E-06	−2.2	9.08E-06
TFDP1	Transcription factor Dp-1	NM_007111.4	−4.1	4.9E-06	−2.6	3.83E-06
TTK	TTK protein kinase	NM_003318.3	−2.4	3.75E-05	−2.2	4.44E-07

a *p*-value calculated using nSolver default settings.

**TABLE 4:** Mutually regulated differentially expressed genes (DEGs) associated with PI3K pathway following 16 μM treatment of MS13 in DU 145 and PC-3 cells at 24 h.

Gene symbol	Gene name	Accession	DU 145		PC-3	
			Fold-change	*p*-value	Fold-change	*p*-value
HSP90B1	Heat shock protein 90 kDa beta (Grp94), member 1	NM_003299.1	2.3	2.9E-05	2.5	9.62E-05
IRS1	Insulin receptor substrate 1	NM_005544.2	−4.9	0.000507	−2.9	0.000286
ITGA3	Integrin, alpha 3 (antigen CD49C, alpha 3 subunit of VLA-3 receptor)	NM_005501.2	−3.3	1.2E-09	−2.1	2.47E-06
ITGA6	Integrin, alpha 6	NM_000210.1	−2.1	6.35E-06	−2.4	6.1E-07
LAMA3	Laminin, alpha 3	NM_000227.3	−2.7	1E-04	−2.0	8.59E-05
PGF	Placental growth factor	NM_002632.5	22.0	4.9E-05	8.9	0.00047
PPP2CB	Protein phosphatase 2, catalytic subunit, beta isozyme	NM_001009552.1	2.3	2.65E-07	3.3	1.31E-08

## Discussion

Based on the results, MS13 has shown to decrease the percentage of viable cells in both DU 145 and PC-3 in a dose-dependent manner. The EC_50_ values of MS13 for PC-3 and DU 145 cells were 7.80 ± 0.7 μM and 7.57 ± 0.2 μM, respectively, much lower than curcumin. Overall, these results are in agreement with previously published data (Lin et al., 2009; Suarez et al., 2010; Citalingam et al., 2015). Besides, MS13 exhibits lower toxicity and highly selective towards PCa cells than normal cells, as indicated by SX values. MS13 showed better cytotoxic activity compared to the parent compound, curcumin against human normal cells, which was consistent with the data described previously (Citalingam et al., 2015; Paulraj et al., 2015; Ismail et al., 2020; Lee et al., 2021). An earlier study reported that FLLL11, which has an identical chemical structure as MS13 was less cytotoxic as curcumin towards human normal cell lines of lung fibroblast (WI-38), bladder smooth muscle and mammary epithelial (non-malignant MCF-10A) (Cen et al., 2009). Moreover, MS13 exhibited a greater anti-proliferative activity against both PCa cells over curcumin in a time and dose-dependent manner. In DU 145 cells, significant inhibition of cell proliferation was observed at 12.5 μM, with a gradual reduction at 25 μM onwards for all time points. Similarly, PC-3 treated cells showed a significant decrease of cell viability beginning at 12.5 μM for 24, 48 and 72 h. This suggests that MS13 inhibits cell proliferation of PCa cells at a lower concentration than curcumin in a time- and dose-dependent manner, consistent with a previous study using bromodeoxyuridine (BrdU) cell proliferation assay (Citalingam et al., 2015). Additionally, chemical modification, including deletion of β-diketone moiety and replacement to five-carbon spacer between the two aromatic rings, have resulted in greater cytotoxic and growth inhibitory effects and enhanced MS13 stability and bioavailability (Gupta et al., 2017; Moreira et al., 2020). Also, other features have been linked to increasing the stability and pharmacokinetic profile of MS13 over curcumin, including the two identical diphenyl rings, the symmetric diphenylpentanoids, the monocarbonyl diarylpentanoids scaffold (α, β-unsaturated ketone), specific substitution at the phenyl rings and retains the curcumin’s 3-oxo-1,4-pentadiene linker (Lin et al., 2006; Liang et al., 2009). Previous studies reported that MS13 demonstrated greater growth inhibition at lower EC_50_ value compared to curcumin in multiple cancer cell lines, including colorectal (Cen et al., 2009; Liang et al., 2009), nasopharyngeal (Liang et al., 2009), breast (Lin et al., 2009), gastric (Yoshida et al., 2018) and pancreatic (Friedman et al., 2009) cancer cells.

Apoptosis is closely related to the survival of cancers and has become an important factor in the development of anti-cancer therapeutic agents (An et al., 2019). MS13 has also been reported to be a more potent inducer of apoptosis than curcumin (Lin et al., 2009). Therefore, the apoptosis-inducing ability of MS13 was investigated by using morphological observations and biochemical assays, including caspase-3 activity and Bcl-2 concentration. The morphology analysis revealed that a dense bright green fluorescent cells with the occurrences of distinct morphological features of apoptosis like membrane blebbing, shrinkage of the cells, and chromatin condensation, were observed after 24 h in MS13-treated DU 145, indicated early apoptosis. As the MS13 dose increased, the cells underwent late apoptotic, marked as an increased population of yellowish orange to bright red fluorescence cells, with the yellow beads in the centre, due to increased permeability of PI (Haugland, 2002; Ng et al., 2013). Notably, the morphological observations were consistent with the quantitative data obtained. The percentage of apoptotic cells in treated DU 145 was significantly increased for both doses at 24 h compared to the control, and a similar trend was observed in PC-3 cells. The highest percentage of apoptotic cells was noted at 48 h for DU 145 (68%; 7.57 µM and 72%; 15.14 µM) and PC-3 (61%; 7.8 µM and 80%; 15.60 µM) as the doses increased. A decrease in the number of apoptotic cells and an increase of necrotic cells was noted at 72 h for both doses in these cell lines. The highest apoptotic activity was observed at 48 h with a higher dose of MS13. The morphological and quantitative assessments revealed that MS13 induced apoptosis in a time and dose-dependent manner. Besides, the percentage of necrotic cells remained low in all treatments, thus indicated that MS13 induce apoptosis rather than necrosis.

Caspases play an important in the execution of apoptosis and lead to the characteristic of morphology changes of the cells undergoing apoptosis, including cell shrinkage, chromatin condensation, and DNA fragmentation (Mansoor et al., 2011). Among these apoptotic caspases, caspase-3 is considered the most important executioner of caspases to mediate apoptosis and has become a primary target for cancer treatment (Los et al., 2002). In this present study, a significant increase of caspase-3 activity was noted in treated DU 145 and PC-3 cells at 48 h, both doses compared to the untreated cells. An earlier study reported that FLLL11 induced caspase-3 activity and cleavage of PARP in pancreatic and colorectal cancer cells (Padhye et al., 2010). Recent studies on DAPs also reported that MS13 (Ismail et al., 2020) and B63 (Rajamanickam et al., 2017) induced apoptosis by activating caspase-3 in colon cancer cells. Bcl-2 is an anti-apoptotic protein, and its overexpression correlates with tumorigenesis, while its inhibition has been a promising strategy for cancer treatment (García-Aranda et al., 2018). Overexpression of Bcl-2 is frequently observed in AIPC, resulting in the evasion of apoptosis (Kim et al., 2017). Hence, Bcl-2 protein has been considered an important target for anti-cancer therapeutics (Hata et al., 2015). Our study reported that Bcl-2 cellular protein was reduced significantly in a time- and dose-specific manner. A greater reduction was noted as the dose was increased to 15.14 µM for DU 145 and 15.60 µM for PC-3 cells. In agreement with the recent study, MS13 induced apoptosis by suppressing the Bcl-2 level in colon cancer cells (Ismail et al., 2020). Similar results were reported when a diarylpentanoid, DM-1 treated in melanoma cells and EF24 in hepatocellular carcinoma cells (Liu et al., 2012; Faião-Flores et al., 2013). Besides, studies on multiple human colon cancer cell lines indicated that DAPs induced apoptosis by activating caspase-3 activity (Subramaniam et al., 2008; Selvendiran et al., 2010; Liang et al., 2017; Min et al., 2020) and reducing Bcl-2 protein (Lin et al., 2011; He et al., 2016). These findings suggest that activation of caspase-3 and downregulation of Bcl-2 in treated cells demonstrated the apoptosis-inducing ability of MS13.

Cell migration is a key feature of metastatic progression for cancers (Paul et al., 2017). Herein, we demonstrated the ability of MS13 in exhibiting anti-migratory effects in DU 145 and PC-3 cells. Based on the cell migration photomicrographs, DU 145 and PC-3 cells incubated with approximately 8 μM of MS13 have displayed wide wound gaps between cells compared to untreated cells after 24 h. Wider wound gaps were noted as the dose of MS13 increased to approximately 16 μM for both cell lines at all incubation periods. Meanwhile, the untreated cells filled the entire wound gaps at 24 h for both cell lines. Besides, quantitative data also showed a consistent result, thus indicating anti-migratory effects of MS13. Supportively, FLLL11 and other diarylpentanoid FLLL12 had shown greater anti-migratory activity than curcumin against breast cancer cells (Lin et al., 2009). Also, similar observations were noted when PCa cells were treated with a diarylpentanoid, Ca37 (Luo et al., 2014). Furthermore, other diarylpentanoids, are EF24, and CH-5 have also exhibited an anti-migratory effect on hepatocellular carcinoma and human gastric cancer cell lines, respectively (Zhao et al., 2016; Silva et al., 2018). These findings suggest that MS13 inhibits the migration of AIPC cells in a dose-dependent manner, which may consequently interfere with these cells' metastatic ability.

For gene expression analysis, our results demonstrated that several DEGs are mutually regulated despite the different biological properties of DU 145 and PC-3 cells. PC-3 cells were derived from bone metastasis with high metastatic potential, harbouring wild-type p53, while DU 145 cells were derived from brain metastasis with moderately metastatic harbouring mutant p53. Treatment on DU 145 and PC-3 cells demonstrated that several DEGs were associated with cell cycle-apoptosis and PI3K pathways, as the most significantly impacted pathways indicating the anti-cancer activity of MS13. Despite numerous pathways that may potentially contribute to the progression of PCa, cell cycle-apoptosis and PI3K pathways were the most frequently altered in primary and metastatic prostate cancer (Armenia et al., 2018; Chung et al., 2019).

CCNA2, CCNB1, CDC25B, CDC25C, and CDC7 were downregulated in MS13-treated DU 145 and PC-3 cells. CCNA2 regulates the G1/S and the G2/M checkpoints (Bicknell et al., 2007). In PCa, downregulation of CCNA2 induced the G1 cell cycle arrest, thus inhibited cell proliferation, invasion, and metastasis (Lee et al., 2009; Yang et al., 2020). CCNB1 has a function in controlling the cell cycle at the G2/M phase, and its overexpression promotes cell proliferation, tumour growth and cancer recurrence (Zhuang et al., 2018). CCNB1 regulates undifferentiated metastatic PCa with poor prognosis (Gomez et al., 2007). Curcumin has shown to downregulate CCNB1 expression in PCa (Thangapazham et al., 2008), colon (SU et al., 2006) and lung cancer (Yang et al., 2012; Zhang et al., 2019) which has been linked to G2/M arrest (Yan et al., 2004). This suggests that the downregulation of CCNA2 and CCNB1 by MS13 treatment may inhibit proliferation, invasion, metastasis in AIPC cells. CDC25B and CDC25C are primarily required for entry into mitosis through activation of CDK1–cyclin B, frequently overexpressed in PCa, which leads to metastasis and poor prognosis (Ozen and Ittmann, 2005; Boutros et al., 2007). The CDC25B inhibitors were reported to inhibit pancreatic cancer cell growth by blocking G2/M phase transition (Guo et al., 2004). Meanwhile, the downregulation of CDC25C by curcumin promotes anti-proliferation in colon cancer (SU et al., 2006). Thus, the downregulation of CDC25B and CDC25C by MS13 treatment may suggest its antiproliferative activity in AIPC cells. CDC7 regulates the G1/S transition, and its overexpression is a common occurrence in multiple human cancer*s* (Gad et al., 2019). In PCa, the knockdown of CDC7 expression causes growth arrest and cell death (Montagnoli et al., 2004; Bonte et al., 2008). This suggests that downregulation of CDC7 by MS13 treatment potentially promote apoptosis in AIPC cells.

In addition, SKP2, AKT1, PKMYT1, TTK and PRKDC were downregulated following MS13 treatment in DU 145 and PC-3 cells. SKP2, a critical component of the SCF^Skp2^ ubiquitin ligase complex, is frequently overexpressed in many human cancers (van Duijn and Trapman, 2006; Wang et al., 2012). SKP2 overexpression contributed to tumorigenesis in PCa (van Duijn and Trapman, 2006; Wang et al., 2012). The downregulation of SKP2 by curcumin treatment inhibited tumour growth, migration, and invasion in multiple cancers, including PCa (Wang et al., 2015; Ding et al., 2017; Khan et al., 2018). This suggests that the downregulation of SKP2 may inhibit cancer growth, migration, and invasion in AIPC cells. AKT1 is a serine-threonine protein kinase, where its downregulation inhibited cell proliferation, induced loss of cell adhesion and subsequent apoptosis, and inhibited cell migration in PCa cells (Cariaga-Martinez et al., 2013). Curcumin inhibited AKT1 resulting in the inhibition of cell growth and cell migration in PCa cells (Chaudhary and Hruska, 2003; Le Page et al., 2006). This may suggest that the downregulation of AKT1 inhibiting cell proliferation and migration in AIPC cells. PKMYT1 codifies a member of the serine/threonine protein kinase family, which is important in regulating the cell cycle through the inactivation of CDKs (Long et al., 2020). PKMYT1 is highly expressed in multiple cancer cells, including gastric, lung and colorectal cancer, which correlates with poor prognosis and disease progression (Asquith et al., 2020). In agreement with the previous finding, downregulation of PKMYT1 was shown to inhibit cell growth and migration (Wang et al., 2020). This suggests the knockdown of PKMYT1 significantly inhibited the growth and migration capabilities of AIPC cells. TTK plays an important role in cell division, and its expression is higher in many human malignancies. Silencing of TTK in PCa, gastric and pancreatic cancer cells inhibited cell proliferation, invasion and migration, and increased apoptosis (Kaistha et al., 2014; Chen et al., 2019; Huang et al., 2020). Thus, it suggests that the inhibition of TTK by MS13 treatment may inhibit cell proliferation and induce apoptosis in AIPC cells. PRKDC is a critical component of DNA repair machinery, and its expression is implicated in tumour progression, metastasis and migration (Yin et al., 2020). The PRKDC knockdown has been reported to attenuate cell proliferation and metastasis in PCa cells (Goodwin et al., 2015; Zhang et al., 2017). Thus, this suggests that the downregulation of PRKDC by MS13 treatment may inhibit cell proliferation and metastasis in AIPC cells.

Besides, MS13 treatment in DU 145 and PC-3 cells has shown the downregulation of PPP3CB, TFDP1, SMAD3, and ENDOG, which the anti-cancer activity have not been reported in PCa but noted in other cancers. PPP3CB, which plays an essential role in the transduction of intracellular Ca^2+^-mediated signals and may has a pro-tumorigenic role in PCa (Brun and Godbout, 2016). PPP3CB knockdown has decreased cell growth in neuroblastoma cells (Shakhova et al., 2019). This suggests the growth inhibition capabilities in AIPC cells by MS13 treatment. TFDP1 is a heterodimerization partner for members of the E2F family of transcription factors. E2F1/TFDP1 forms a complex involved in cell cycle progression (Abba et al., 2007). In agreement with our findings, the downregulation of TFDP1 may inhibit cell growth, as reported in hepatocellular carcinoma (Yasui et al., 2003; Zhang et al., 2013). Thus, the downregulation of this gene by MS13 treatment potentially inhibits cell growth in AIPC cells. SMAD3 encodes for a key regulator in the TGF-β pathway that activates or represses gene transcription. This gene is highly expressed in PCa (Lu et al., 2007; Xia et al., 2014). Inhibition of SMAD3 inhibited cancer growth, invasion and metastasis in lung and melanoma cancer (Tang et al., 2017). This suggests the downregulation of SMAD3 by MS13 treatment may inhibit cancer growth and metastasis in AIPC cells. ENDOG encodes a mitochondrial protein that plays a major function in apoptosis. Despite its function as an apoptosis regulator, ENDOG has dual roles in cell death and survivals (Huang et al., 2006; Büttner et al., 2007). Inhibition of ENDOG resulted in the decreased cell viability, cell distribution changes in cell cycle phases, and reduced cell proliferation, suggesting its oncogenic properties (Huang et al., 2006). Moreover, ENDOG overexpression in colorectal and gastric cancer cells associated with tumour development (Yoo et al., 2008). Thus, the downregulation of ENDOG by MS13 may suggest its anti-proliferation properties in AIPC cells.

On the other hand, multiple genes, including CDKN1A, PIK3R3, GADD45B and GADD45A, were upregulated when exposed to MS13 treatment. CDKN1A acts as a potent cyclin-dependent kinase inhibitor, whereby its inhibition in PCa leads to cell cycle progression inhibition at G1 and induced G2 arrest (Baretton et al., 1999; Li et al., 2017). Curcumin treatment upregulated CDKN1A in PCa and lung cancer cells, resulting in the induction of cell cycle arrest and apoptosis (Srivastava et al., 2007; Zhang et al., 2019). This suggests that the upregulation of CDKN1A by MS13 treatment may induce apoptosis in AIPC cells. PIK3R3 is involved in regulating phosphatidylinositol 3-kinase activity and overexpressed in some types of cancers, such as colorectal, lung, and gastric cancer (Zhou et al., 2012; Yu et al., 2015; Cai et al., 2016). However, PIK3R3 overexpression promotes chemotherapeutic sensitivity and apoptosis in colorectal cancer cells (Ibrahim et al., 2018). Our data suggest that upregulation of PIK3R3 by MS13 treatment may induce apoptosis in AIPC cells. GADD45B is a tumour suppressor gene that functions as an inhibitor of cell growth. Upregulation of GADD45B in PCa contributed to cell growth inhibition and apoptosis induction (Zhang et al., 2017). An induced expression of GADD45B was noted in PCa cells treated with matrine, a naturally occurring alkaloid that resulted in inhibition of cell proliferation, migration, invasion and promoting cell death (Huang et al., 2018). GADD45A, which play roles in apoptosis and cell cycle arrest, were found downregulated in many malignancies (Tamura et al., 2012). However, the upregulation of GADD45A may subsequently inhibit the G2/M transition of the cell cycle and induces apoptosis in PCa (Liebermann and Hoffman, 2011; User and Muyan, 2015). In lung cancer, curcumin treatment increased GADD45A expression, which leads to the reduction of cell proliferation rate (Saha et al., 2010). Thus, this suggests that the upregulation of GADD45B and GADD45A by MS13 treatment may inhibit cell proliferation and promote apoptosis in AIPC cells.

MS13 treatment on DU 145 and PC-3 cells also led to the downregulation of several mutually regulated DEGs associated with PI3K pathway, including IRS1, ITGA3, ITGA6, and LAMA3. IRS1 is the principal effector of the insulin and insulin-like growth factor signalling pathways, by which its expression often linked to tumorigenesis and radioresistance (White et al., 1985; Gorgisen et al., 2019). Upregulation of IRS1 increases tumour proliferation and metastasis in many human malignancies, including PCa and breast cancer cells (Chang et al., 2002; Byron et al., 2006; Reiss et al., 2012). However, silencing of IRS1 with rapamycin treatment inhibited the growth of PCa xenografts (Oliveira et al., 2008). Hence, this study suggests that downregulation of IRS1 indicates the role of MS13 in inhibiting cell proliferation, metastasis and induce apoptosis in AIPC cells. ITGA3 is a cell surface adhesion protein that interacts with extracellular matrix proteins, and its expression correlated with cancer metastasis (Jiao et al., 2019). ITGA3 is involved in cell proliferation, migration, and invasion in many cancers, including gastric, non-small cell lung, PCa and colorectal cancer, by activating the PI3K-Akt signalling pathway (Chen et al., 2018; Jiao et al., 2019). Curcumin has been reported to significantly inhibited cell proliferation and invasion and induced death in lung cancer cells by downregulating ITGA3 (Li et al., 2017). Therefore, the downregulation of ITGA3 by MS13 treatment may contribute to the induction of apoptosis as well as inhibition of cell proliferation, cell migration and invasion in AIPC cells. Another member of the integrin family of proteins, ITGA6, has also been correlated with cell migration and invasion in cancer cells (Golbert et al., 2013; Brooks et al., 2016; Hu et al., 2016). Upregulation of ITGA6 was reported in ovarian cancer, leading to multi-drug resistance (Wei et al., 2019). This suggests that downregulation of ITGA6 by MS13 could inhibit cell migration and invasion in AIPC cells. LAMA3 encodes laminins that are essential for the formation of the basement membrane and plays an additional role in regulating cell migration and mechanical signal transduction (Castro et al., 2018). Increased level of LAMA3 was reported in pancreatic ductal adenocarcinoma, ovarian and liver cancer (Svoboda et al., 2017; Zhang et al., 2019; Tang et al., 2019; Yang et al., 2019). LAMA3 also provides instruction for making a subunit of a protein called laminin 332, promoting cell migration in cancer cells (Carpenter et al., 2017; Oh et al., 2017). Therefore, the downregulation of LAMA3 by MS13 may reduce cell migration in AIPC cells.

In contrast to the downregulated genes, several DEGs associated with PI3K pathway, including HSP90B1, PGF, and PPP2CB, were upregulated following MS13 treatment. HSP90B1 is a molecular chaperone protein in which its downregulation is associated with prostate carcinogenesis and metastasis (Ni and Lee, 2007; Howard et al., 2008). Therefore, upregulation of HSP90B1 by MS13 treatment may contribute to the inhibition of cancer growth and metastasis in AIPC. PGF is a member of the vascular endothelial growth factor (VEGF) family. Its overexpression was shown to inhibit *in vivo* tumour growth, angiogenesis, and metastasis of lung, brain, and colon tumours (Xu et al., 2006). Thus, the upregulation of PGF by MS13 treatment may inhibit tumour and angiogenesis in AIPC. PPP2CB is a serine/threonine-protein phosphatase 2A catalytic subunit beta isoform, and it is implicated in the negative control of cell growth and division. In PCa, downregulation of PPP2CB is associated with poor outcomes and contributes to the aggressiveness of disease (Wissmann et al., 2003; Bott et al., 2017). Hence, this suggests that the upregulation of PPP2CB by MS13 treatment could inhibit cell growth in AIPC.

## Conclusion

The diarylpentanoid MS13 has demonstrated greater cytotoxicity, growth inhibitory and anti-migratory effects on androgen-independent prostate cancer cells than the parent compound, curcumin. In addition, MS13 mediates apoptosis confirmed by morphological observation, increased caspase-3 activity, and reduced Bcl-2 levels. The gene expression study revealed that mutually regulated DEGs were associated with cell cycle-apoptosis and PI3K pathways, as the most significant pathways targeted by MS13. Thus, our findings suggest that MS13 may demonstrate the anti-cancer activity by modulating DEGs associated with the cell cycle-apoptosis and PI3K pathways, thus inhibiting cell proliferation and cell migration and inducing apoptosis in AIPC cells.

## Data Availability

The authors acknowledge that the data presented in this study must be deposited and made publicly available in an acceptable repository, prior to publication. Frontiers cannot accept a article that does not adhere to our open data policies.
